# Cases of Malignant Pneumonia

**Published:** 1849

**Authors:** Edward R. Parks


THE NORTH-WESTERN

### MEDICAL AND SURGICAL JOURNAL.

Vol. I.] FEBRUARY AND MARCH, 1849. [No. 6.

### Part I.—Original Communications.

### ARTICLE I.

*Cases of Malignant Pneumonia, with remarks.* Read before the Kosciusko county Medical Society. By EDWARD R. PARKS, M.D.

Malignant Pneumonia is a disease of no small interest to the physician, especially the physician of the west. The suddenness of its invasion, the deceptive character of its symptoms, and the rapid course and very frequently fatal termination of the disease, must at once impress the mind of every physician with the importance of being well acquainted with its symptoms, its astonishingly rapid course, and the plan of treatment most likely to prevent its generally fatal termination.

Although this disease has occasionally prevailed in various portions of the United States since 1806, and sometimes in the form of a wide spread and desolating epidemic, yet it is a lamentable fact that even now we know of no good account of the disease in any standard work in the hands of the profession.

The best account that we have seen is to be found in Watson's Practice of Physic, in a note by the American Editor, (T. Francis Condie,) page 611.

Some of the common names for this disease have been "head pleurisy" and "the cold plague." By physicians it has been called, but in our opinion with doubtful propriety, "typhoid pneumonia." We have heretofore described a variety of pneumonic disease that we denominated "typhoid pneumonia," but in this form of disease the symptoms differ so widely from an ordinary typhoid case, that we have taken the liberty to call it malignant pneumonia. In fact the title pneumonia is itself somewhat objectionable, from the fact that in some instances there are no symptoms that would lead to suspect thoracic disease. Notwithstanding this is the case in some instances where the disease runs a rapid course and terminates fatally in a few hours, yet we believe there are no cases in which the disease lasts for several days that do not develop symptoms of lung disease. The first appearance of the disease in this country, that came under notice was in the latter part of the spring of 1845. From the great diversity of symptoms presented in different cases of this malady, arises the extreme difficulty that exists in describing its symptoms in an intelligible manner. Perhaps we can give the clearest idea of the disease by referring to the following.

Case I.—W. E., æt. 15, a lad of tolerably good constitution, was attacked about the first of April, 1845. We saw him first at ten o'clock, P.M. He then complained of a dull heavy pain in the head, with some lassitude, &c. There was little arterial disturbance; no fever. He was bled to, perhaps, ten ounces, and ordered to take a portion of compound cathartic pills. The next morning we received a summons very early to visit him. When we arrived, the father gave us the following account: The pills were taken in a few moments after we left, but were ejected. Shortly afterwards he thought the patient fell asleep, as he heard nothing of him till morning, when he was found in a state of insensibility. We found the patient in a state of insensibility, with the eyes very prominent and staring. There was nothing very remarkable in the appearance of the pupils. The extremities and head were in incessant convulsive motion; pulse a little fuller and more frequent than usual; extremities cold, and the whole surface of the body decidedly moist, though the head and breast were somewhat hot. The features were frightfully distorted; the respiration was hurried and loud, and there was occasional vomiting. It was impossible to get the patient to swallow anything whatever. We abstracted a pint of blood, administered an enemata of oleum terebinth., and applied sinapisms to the ankles and wrists along the whole length of the spine, and over the epigastrium. The condition of the patient grew worse, and he died about three or four o'clock of the same afternoon.

Case II.—Mrs. L., the mother of the young man whose case we have just related, æt. 33, of spare habit and delicate constitution, complained, perhaps an hour before the death of her son, of some pain in the head, lassitude, etc., and requested us to prescribe for her, though she thought her symptoms probably depended upon fatigue and anxiety about her son. We prescribed an emetic of ipecac., which operated mildly. An hour afterwards, she expressed herself as feeling quite comfortable. In less than an hour after this we were summoned to visit her in haste. We set off immediately on our way, which was not over a half mile. On our arrival we found the patient delirious, and in a state of the most perfect restlessness; eyes prominent and imploring, and the whole surface of the body bathed in a copious and very offensive perspiration, with an inability to swallow anything. She was bled, at two bleedings within an hour, about a quart, and sinapisms were extensively applied. Her condition grew decidedly worse, with aggravation of all her symptoms; her extremities soon became icy cold, and she died about twelve o'clock that night. Drs. R. M. Kendall, Wm. Parks, and my brother, J. F. Parks, saw this patient. The two foregoing were the first cases of this description that we had ever witnessed.

Case III.—J. R., æt. 40, a respectable farmer, of good habits and constitution, was attacked about the tenth of April, 1845. He had eaten his supper and gone to bed as well as usual, but awoke about midnight, complaining of feeling cold and chilly, and requested one of the family to put more clothes over him. He complained of lassitude and pain in every part of his body. In an hour afterwards, we found him quite delirious and writhing with pain; talking incoherently and incessantly; bathed in a copious and very offensive perspiration; with constant jactitation; pulse tolerably full, vibrating and inelastic; tongue covered with a whitish tenacious phlegm; dysuræ amounting in fact to complete retention, or rather suppression, and considerable dyspnœa. Our prescription was—℞. disulphate of quinine, ten grains; sulphate of morphine, half a grain, every half hour, and sinapisms to the extremities, to the spine, and over the epigastrium. Under this treatment, the condition of the patient rapidly improved. The delirium, jactitation, and sweating subsided, and, when we saw the patient twelve hours after the attack, all the most alarming symptoms had disappeared. He now had some cough, and complained of pain in the right side of the chest, for which we ordered a blister to be laid on the affected part and gave twenty grains of the submur. hyd. to be followed with a full dose of oleum ricini in two hours. The other medicines were continued, but given less frequently; the powders of quinine and morphine every two hours, the brandy every hour, with orders to discontinue the brandy in case the skin became hot and dry. The next morning the condition of the patient was much improved; the cathartic had operated well; the cough and pain in the side had subsided; the patient had rested pretty well through the night; and the strangury, which had been treated with demulcent drinks, and warm fomentations over the hypogastric region, was completely relieved. The powders were continued, though in smaller quantities; convalescence soon ensued, and the patient had a rapid and perfect recovery.

CASE IV.—Mrs. T. H. æt. 25, a married woman of good constitution, was attacked on the 19th of April, 1845, about sunset, with chilly sensations, aching of the limbs, and pain in the head. We found her, about an hour after the attack, with the extremities very cold; the pulse very small and irregular; face flushed and hot; features distorted; delirious, and giving no attention to anything said to her; with her head constantly in motion. We prescribed, ℞—disulphate of quinine ten grains; sulphate of morphine, half a grain every two hours, with a table spoonful of brandy every half hour, sinapisms to be applied as in the foregoing cases, with a dry warmth to the extremities. Twelve hours afterwards we found the patient rational, the feet and hands warm, and the restlessness relieved with pulse fuller though irregular and intermittent, and complained of pain in the head. Continued brandy and powders as before, with the addition of five grains of the sub. mur. hyd. to each powder. This was in the morning, the patient was ordered to take oleum ricini one ounce, oleum terebinth. half an ounce, that evening. We saw the patient early next morning, the bowels had been evacuated freely; she had a slight fever, was somewhat restless, complained of some pain in the head and chest, had a cough, and complained of some tenderness of the gums. Disulphate of quinine, three grains; sulph. morphine, pulv. ipecac. aa, half a grain to be taken every three hours. A blister was laid on the chest over the site of pain. Brandy discontinued. Under this treatment she gradually recovered, though she was considerably ptyalized which somewhat protracted the convalescence.

CASE V.—J. C. P., æt. 23, a gentleman of good habits, was attacked on the morning of the seventh of March, 1848. He awoke from sleep with chilly sensations, aching of the limbs, etc. We found him about an hour afterwards, with cold extremities, the pulse small and irregular, respiration short and difficult, with a peculiar diffuse livid flush on the face. He complained of severe pain in the head, and throughout the entire chest; he could but very partially inflate the chest, and this was attended with severe pain. He had a constant desire to cough, but the effort was almost entirely suppressed. Disulphate of quinine, five grains; sulph. morphine, one grain, this to be taken every two hours. Sinapisms were applied to the ankles and wrists, and also dry warmth. Saw the patient at eight o'clock P. M. Found him tolerably comfortable. The extremities were warm, head entirely relieved, some pain yet remained in the chest, the powders were continued one every three hours. Saw the patient next day at noon—he felt quite smart. As the bowels had not been evacuated for some forty-eight hours, we ordered him to take sulph. magnesia and senna, and three powders of quinine five grains each, the next forenoon. His recovery proceeded rapidly from this time without a solitary unpleasant symptom.

*Causes*—The predisposing cause to this disease is undoubtedly malaria, whilst the exciting cause depends upon sudden vicissitudes connected with some peculiar condition of the atmosphere. We cannot, however, dwell upon these points.

*Pathology*—Various opinions have been entertained by the profession with reference to the proximate cause of this disease. By some it has been supposed to consist in inflammation either of the brain or lungs, or both of these organs; while others insisted upon its depending upon congestion. It appears evident to us that the primary disease is located in the nervous system, and that congestion and inflammation are secondary results. That inflammation does take place in some instances cannot be doubted, but that it never does in the early stages, is equally clear. That there could have been no inflammation at any time in the fifth case reported in this paper we think is sufficiently clear from the rapid recovery that ensued, although the early symptoms were of the severest character.

*Diagnosis*—There is but one disease that malignant pneumonia is liable to be confounded with, and that is inflammation of the brain. The sudden invasion of the attack, the great prostration, extreme jactitation, the condition of the extremities, the almost total want of reaction, and the feeble, irregular, and flickering pulse, are amongst the most important symptoms indicative of this disease.

The nature and type of prevailing diseases will also afford much important information.

Indeed this is, perhaps, the most important consideration in forming a correct diagnosis in the malady. Should pneumoniac affections of a typhoid type be extensively prevailing, and any doubt about the character of the disease remains, this circumstance may fairly settle the question.

